# Severe bullous cutaneous anthrax with malignant edema

**DOI:** 10.1590/0037-8682-0164-2021

**Published:** 2021-04-28

**Authors:** Ayşe Sağmak Tartar, Ayhan Akbulut, Betül Demir

**Affiliations:** 1 Firat University, Faculty of Medicine, Department of Infectious Diseases and Clinical Microbiology, Elazığ, Turkey.; 2 Firat University, Faculty of Medicine, Department of Dermatology, Elazığ, Turkey.

Anthrax plays an important role in the history of infectious diseases. Although the frequency of cases has decreased in Turkey, anthrax remains an endemic disease^1^ ^,^ ^2^. A 55-year-old man noticed a painless wound on the dorsal side of his hand, after having skinned and carved a lamb one week previously.

**FIGURE 1:**
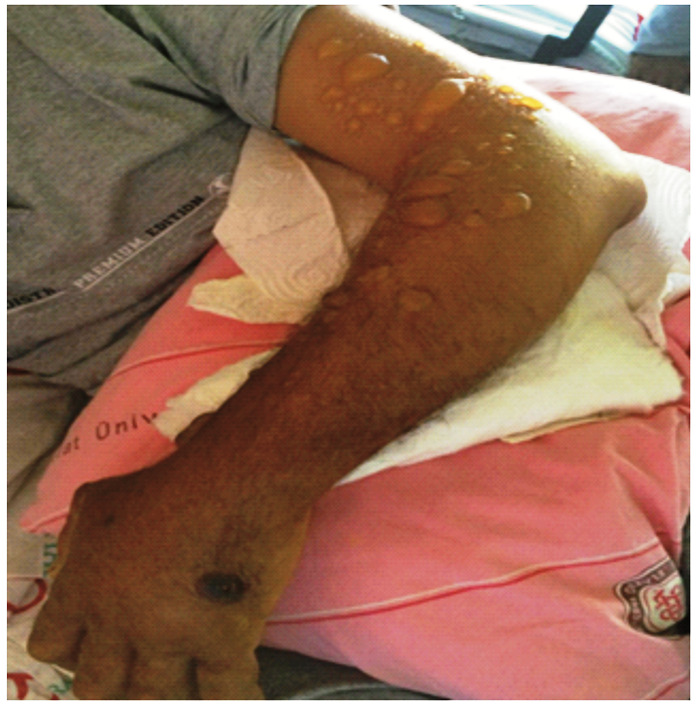
Multiple bullous lesions seen on day 2 of treatment.

**FIGURE 2:**
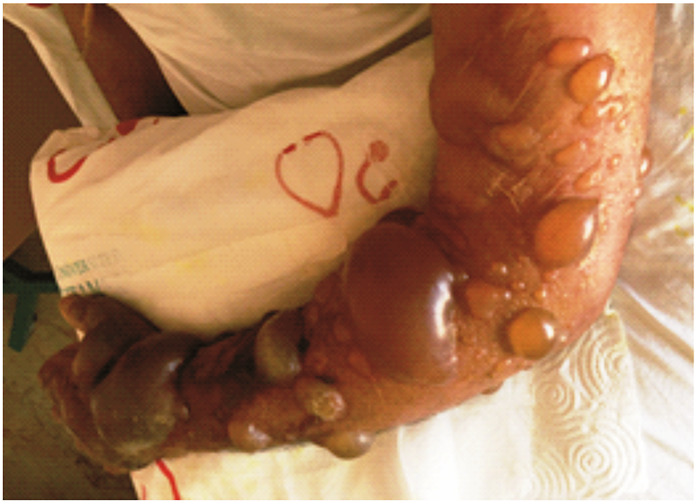
Appearance of the bullous lesions on day 7 of the treatment.

**FIGURE 3:**
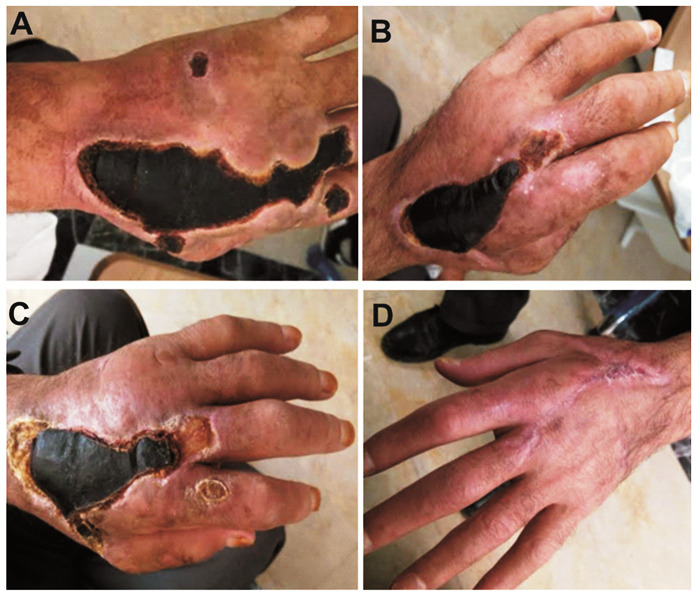
Appearance of the lesions at (**A)** 1 month, (**B)** 2 months, (**C)** 3 months, and (**D)** 4 months of follow-up.

Upon physical examination, a black, 2-cm ulcerative lesion with no edema was observed on the dorsum of his right hand. An aspirate sample from the ulcerative lesion was cultured, and numerous encapsulated gram-positive bacilli, identified as *Bacillus anthracis* were seen; the isolates were sensitive to penicillin. On the second day of treatment with penicillin, bullae of several sizes developed around the circumference of the lesion (Figure 1). Lymphangitis developed in the same arm, and methylprednisolone was added to the treatment regimen. However, the patient developed respiratory distress. Posteroanterior chest radiography scans revealed that the trachea was deviated to the left. This might have occurred because of the possible development of serious edema, especially in lesions around the face and neck. The bullous lesions enlarged and spread over the entire arm (Figure 2). Subsequently, some bullae burst, and typical anthrax pustules ensued. The appearance of the lesions at 1, 2, 3, and 4 months of follow-up are shown in Figure 3A, B, C, and D, respectively.

 This case of serious bullous lesions that subsequently had a severe clinical course was remarkable. Patients suspected of having cutaneous anthrax should be hospitalized, treated, and followed up closely for sepsis and other systemic complications.
